# Soil Suppressiveness Against Pythium ultimum and Rhizoctonia solani in Two Land Management Systems and Eleven Soil Health Treatments

**DOI:** 10.1007/s00248-023-02215-9

**Published:** 2023-03-31

**Authors:** Viola Kurm, Johnny Visser, Mirjam Schilder, Els Nijhuis, Joeke Postma, Gerard Korthals

**Affiliations:** 1 Wageningen University and Research, Biointeractions and Plant Health, P.O. Box 16, 6700 AA Wageningen, The Netherlands; 2 Wageningen University and Research, Field Crops, Edelhertweg 1, 8219 PH Lelystad, The Netherlands

**Keywords:** Soil health treatments, Disease suppressiveness, Soil microbiome, *Pythium ultimum*, *Rhizoctonia solani*

## Abstract

The soil microbiome is known to be crucial for the control of soil-borne plant diseases. However, there is still little knowledge on how to modify the soil microbiome to induce or increase disease suppressiveness. In the present study, we applied eleven soil health treatments combined with conventional and organic agricultural management in a long-term field experiment. Suppressiveness against *Pythium ultimum* and *Rhizoctonia solani* was assessed in bioassays for 2 years. In addition, the microbiome community composition and microbial abundance were determined. We found that while several treatments changed the microbial community composition compared to the control, only a combination treatment of anaerobic soil disinfestation, hair meal, and compost addition resulted in suppressiveness against *P. ultimum*. *Pythium* suppressiveness is likely to have been caused by an increased microbial abundance and activity. Moreover, the increased abundance of several bacterial taxa, such as *Pseudomonas* sp., *Chryseobacterium* sp., members of the family Chitinophagaceae, and the fungal genus *Mortierella* sp. and family Trichosporonaceae, was measured. There was no overall difference in suppressiveness between conventional and organic land management. Also, no suppressiveness against *R. solani* could be detected. Our results indicate that a treatment combining the reduction of microorganisms followed by a recovery phase with high amounts of organic amendments may be more effective in inducing suppressiveness than treatments consisting of only one of these measures.

**Supplementary Information:**

The online version contains supplementary material available at 10.1007/s00248-023-02215-9.

## Introduction

Soil health and how to improve it has become one of the biggest challenges of recent years. There is a demand for a reduction of artificial fertilizers and chemical pesticides, while maintaining a high agricultural productivity. This goal can only be achieved by healthy soils in which crops are provided with the necessary nutrients and are protected from diseases. Assessing chemical and physical soil parameters as an indicator for soil health has become increasingly common. In addition, in recent years, interest in soil biological parameters and especially soil microorganisms has increased. Soil microorganisms are known to be involved in soil nutrient mineralization and the suppression of diseases. But even though the role of microorganisms has been well determined, our knowledge on how to manipulate microbes to our benefit remains scarce.

One important role of soil microorganisms is the mitigation of plant diseases. Soil microbial community composition and specific microbial taxa have been shown to have direct effects on the crop by promoting plant growth and inducing plant resistance against seed and soilborne pathogens [1]. In addition, the soil microbiome is involved in the development of disease-suppressive soils. Disease-suppressive soils are defined as soils in which diseases either cannot establish or even if present cause no or less damage to the crop [2]. Soil suppressiveness is commonly divided into general and specific suppressiveness [3]. General suppressiveness is associated with a high activity and abundance of soil microorganisms, which compete with and thereby suppress the pathogen. As such, general suppressiveness can often be improved by organic amendments that increase soil microbial biomass [4]. Also, a high microbial diversity and thus functional redundancy is thought to contribute to general suppressiveness [5]. General suppressiveness is usually effective against a broad range of pathogens, among which suppressiveness against the oomycete *Pythium ultimum*. On the other hand, specific suppressiveness relies on the action of specific fungal or bacterial species or even strains, which have the ability to antagonize a specific pathogen. Specific suppression has been suggested for several pathogens, such as *Fusarium oxysporum, Rhizoctonia solani*, or *Streptomyces scabies* [6]. Soil suppressiveness is often a combination of both general and specific suppressiveness.

The role of soil microorganisms in disease suppressiveness is now well characterized, but it remains to be determined how to influence the microbiome to create and improve suppressiveness. A number of studies have found that organic land management systems often have stronger disease-suppressive abilities than conventional systems [7, 8]. This is assumed to be due to higher microbial diversity and activity [9]. Moreover, several soil treatments in the form of amendments or disinfestation practices have been tested for their effect on disease suppressiveness. For example, amendments with chitin and keratin-containing compounds have been demonstrated to increase soil suppressiveness against a fungal pathogen by increasing the population of chitin- and keratinolytic bacteria [10]. The addition of organic amendments, such as composts, has been shown to alter the soil microbiome composition and increase microbiological biomass and activity increasing suppressiveness [11]. A similar effect was found for cover crops [12, 13]. Finally, soil disinfestation practices have often been demonstrated to lead to persisting changes in the soil microbiome and thereby disease suppression [12]. These studies show the high potential of a range of soil health treatments for improving disease suppressiveness.

While the addition of organic amendments, cover crops, and soil disinfestation has been shown to increase disease suppressiveness in some studies, the effects are not consistent between studies or over years due to a variability in biotic and abiotic factors. Partly, the inconsistency in results between studies stems from assessing the effect of land management systems and soil treatments in a large number of different soils, climates and therefore under widely differing conditions. There is a shortage of studies comparing soil health treatments at a common location as well as of studies combining land management practices with additional soil health treatments. In addition, most studies measure rather short-term effects of single treatment applications. Still, we hypothesize that soil management and some types of soil health treatments may have an effect of soil suppressiveness against several soil pathogens. In order to advance current knowledge, we used a long-term field experiment, which combined organic and conventional land management with eleven different soil health treatments. In this study, we measured disease incidence after the introduction of a pathogen in a bioassay as an indication of soil suppressiveness in 2 consecutive years as well as soil microbial abundances and soil community composition. We aimed to assess and compare the effects of agricultural practices and soil health treatments on disease suppressiveness against the soil-borne pathogens *Pythium ultimum* and *Rhizoctonia solani*. In order, to determine the longevity of the treatment effects, suppressiveness was measured in the same year as the application of the soil health treatments and in 1 consecutive year. In addition, we investigated if suppressiveness is associated with changes in soil microbial biomass and community composition.

## Material and Methods

### Long-Term Field Experiment

The long-term field experiment, called “Soil Health Experiment”, has been established in 2006 in Vredepeel, The Netherlands (51° 32′ 27.10″ N and 5° 51′ 14.86″ E), on sandy soil with a history of use as an arable field. Specifically, the soil represent a Gleyic Podzol with a low clay content and sensitivity to drought. For mean annual climate data, see Table S1. The experiment was divided into main plots under organic or conventional management and 10 subplots with different soil health treatments (Table 1). The experiment is set up with four replicates per combination in a split-plot block design. Each plot had a size of 6×6 m. The soil health treatments were applied in July 2006, 2009, and 2018, while conventional and organic management were maintained since 2006 [14]. From 2006 to 2016, the following crop rotation was followed: cereal (wheat in conventional, barley in organic), potato, lily, cereal (wheat in conventional, barley in organic), potato, carrot, maize (2012, 2013, 2014), peas, and cereal (wheat in conventional, barley in organic). In 2017, potato was planted followed by Japanese oat as a cover crop from September until December. In 2018, peas were planted. In 2019, in spring, barley and peas were cultivated as a cover crop followed by leek as a main crop. In 2020, summer barley was planted followed by the cover crop fodder radish from August until November. The organic fields were fertilized using only organic fertilizers. In the conventional system, organic fertilizers were complemented with N, P, and K as artificial fertilizer. For the complete fertilization scheme, see Table 2. Both systems were tilled 25 cm deep. There were no other differences between the organic and conventional management system.

**Table 1:** Description of the 11 treatments, treatment code, and description

Treatment	Code	Description
Fallow control	CTR	No treatment, only weeding
Grass clover cover crop	GCC	Cultivation of 20% white clover (*Trifolium repens*) and 80% English ryegrass (*Lolium perenne*) from July to November and incorporation into the upper soil layer (0–20 cm)
*Tagetes patula* cover crop	TAG	Cultivation of *Tagetes patula* from July to November and incorporation into the upper soil layer (0–20 cm)
Cover crop mix	MIX	Cultivation of the following mixture: *Trifolium alexandrinum* (11%), *Fagopyrum esculentum* (5%), *Brassica oleracea/* kale (3%), *Linum usitatissimum* (3%), *Raphanus sativus* subsp. *oleiferus* (1%), *Trifolium resupinatum* (23%), *Phacelia tanacetifolia* (6%), *Avena strigosa* (3%), *Ornithopus sativus* (19%), *Helianthus annuus* (1%), *Lolium multiflorum* (18%), *Brassica napus* (2%), *Brassica napus*/ rapeseed (3%), *Vicia villosa* (2%) from July to November and incorporation into the upper soil layer (0-20 cm)
Compost	CMP	Amendment of each subplot with 50 t/ha mature compost and incorporation of 20 cm into the soil surface The compost consisted of green waste (pruning waste and cuttings) and was produced by Attero (Maastricht, The Netherlands)
Chitin	CHI	Amendment of each subplot with 20 t/ha shrimp debris (Gembri) and incorporation of 20 cm into the soil surface
Hair meal	HRM	Amendment of each subplot with 7 t/ha hair meal (from pig hair) and incorporation of 20 cm into the soil surface
Anaerobic soil disinfestation	ASD	Amendment of each subplot with 180 kg (50 t/ha) fresh grass, watering with 20 mm water, and airtight sealing with plastic (HyTibarrier foil). The foil was removed after 8 weeks
Soil disinfestation	CSD/OSD	In the conventional system, chemical wet disinfestation (CSD) was done by injecting 300 l/ha Monam© (active ingredient metam-sodium) and covering the soil with TIF foil In the organic system, disinfestation with seed meal (OSD) was done by amendment with 25 kg (0.7 t/ha) Terrafit-Biofum (defatted seed-meal of seeds of brown mustard *Brassica juncea*) and sealing with plastic (TIF foil)
Combination treatment	AHC	Combination of the anaerobic soil disinfestation, hair meal, and compost treatment. After the anaerobic soil disinfestation, 7 t/ha hair meal and 50 t/ha compost were added

**Table 2:** Fertilization scheme in the organic and conventional management system. No additional P was added as a chemical fertilizer

Year/crop	System	Solid cattle manure (m3/ha)	Cattle slurry (m3/ha)	Vinassekali (m^3^/ha)	Artificial fertilizer N	Artificial fertilizer K_2_O (kg/ha)	Total active N (kg/ha)	Total active P (kg/ha)	Total active K (kg/ha)
2017/potato	Organic	25	25	-	-	-	112	96	278
	Conventional	-	40	-	110	-	209	42	188
2018/pea	Organic	-	22	-	-	-	57	34	90
	Conventional	-	22	-	-	-	57	34	90
2019/leek	Organic	-	25+40^1^	4	-	-	238 (173)^2^	100 (67)^2^	500 (386)^2^
	Conventional	-	25+40^1^	-	110	75	278 (214)^2^	89 (55)^2^	322 (207)^2^
2020/barley	Organic	-	30+25^3^	-	-	-	78^4^	41^4^	147^4^
	Conventional	-	0+25^3^	-	90	90	90^4^		90^4^

^1^25 m^3^/ha applied in March before te cover crop, 40 m^3^/ha applied in June before the cultivation of leek.

^2^Amount of active N, P, and K without the spring application of 25 m^3^/ha cattle slurry.

^3^In the organic system, cattle slurry was applied before the cultivation of summer barley. In both systems, 25 m^3^/ha cattle slurry was applied after harvest.

^4^Amount of active N, P, and K before the cultivation of summer barley.

### Soil Sampling

**Fig. 1:**
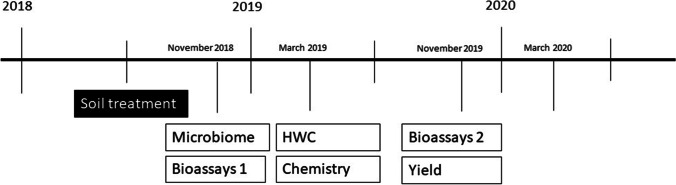
Timeline of application of the 11 soil treatments and sampling times

In November 2018, soil samples were taken for assessing the soil microbiome and for the bioassays (Fig. 1). In March 2019, soil sampling was done for hot water extractable carbon (HWC) measurements and soil chemical properties and in November 2019 soil was sampled again for a second bioassay and crop yield. At all time points, soil was sampled from the upper 25 cm using a 12-mm soil auger, taking several samples per plot that were pooled and mixed. Per subplot, per sampling, 2 to 5 l soil were sampled depending on the total amount needed.

### Soil Chemical Properties

Soil chemical properties were measured in 2019 by Eurofins (Wageningen, The Netherlands) (see Table S2).

### Bioassay

Bioassays were used to estimate the suppressiveness against *Pythium ultimum* and *Rhizoctonia solani* in 2 years after the soil health treatments by assessing disease incidence with and without the addition of the pathogen to the soil. For the bioassays, 2.2 kg of field soil were used from each subplot.

*Pythium ultimum* has been renamed *Globisporangium ultimum* [15]. However, for the sake of continuance, we here use the basionym *Pythium ultimum*. Disease incidence of *P. ultimum* was assessed with garden cress (*Lepidium sativum*) [16]. To half of the soil collected per subplot, 10 g of *P. ultimum* (culture code: Py1, 2005) was cultivated on millet seed for 8 days at 20 °C. Subsequently, the seed was homogenated in a blender and mixed with sand was added to yield 0.25 g *Pythium* per L soil. The other half was not inoculated to assess natural infection. The inoculated and uninoculated soil was mixed in plastic bags and incubated at 20 °C for 2 days. Per subplot, four pots were filled with 95 ml inoculated soil and four pots with uninoculated soil. In each pot, 0.5 g of garden cress seeds was sown. All pots were placed on individual trays to prevent cross-infection between the treatments. The pots were randomized per block and incubated in a climate cell at a 23/18 °C and 16/8-hour day/night cycle at a relative humidity of 60%. In the first 2 days, the pots were covered with plastic foil to prevent evaporation and increase emergence. Seven days after sowing, the percentage of diseased plants per pot was estimated, and the fresh weight of the above-ground plant parts was determined.

Disease incidence of *R. solani* was determined by measuring the spread of the pathogen among sugar beet (*Beta vulgaris*) seedlings [17]. The test was conducted in a climate cell at a 23/18 °C day/night cycle in 4 × 25 cm tanks (1.2 l soil) with an automated watering system keeping the humidity at −50 mbar (pF 1.7). Per tank, 22 sugar beet seeds were seeded in two rows with a distance of 2 cm. After the emergence of the seedlings, the pathogen was added at the beginning of each row. The pathogen *R. solani* AG 2-2IIIB isolate 12-194a was grown on sterilized oat kernels and added 2 cm in front of the seedlings beneath the ground surface. The spread of the disease was assessed twice per week by scoring disease symptoms (distinct brown-gray lesions on the stems at soil level, typical for *R. solani*, wilted, and dead plants). Disease spread 21 days after inoculation in 2018 and 26 days after inoculation in 2019 were used in the analysis, since, in this period, the maximal disease spread was reached in some tanks. Natural infection was assessed as disease spread before inoculation with *R. solani*. Therefore, disease spread after inoculation represents the total disease spread as a sum of natural infection and spread after inoculation.

### Soil Microbiome Quantification and Community Composition

Both soil fungi and soil bacteria were quantified by qPCR in order to assess if the used treatments have an effect on the number of microorganisms and whether this measurement is associated with suppressiveness. DNA was extracted from the soil samples according to the procedure described in Andreo-Jimenez, Schilder, Nijhuis, Beest, Bloem, Visser, Os, Brolsma, Boer, Postma and Stabb [10] using the MagAttract PowerSoil DNA isolation Kit (Qiagen, Germany). In this procedure, the manufacturer’s protocol was adapted to fit an input weight of 1 g of soil for each sample. The resulting DNA eluates stored at −20 °C until further use. DNA concentration was measured with a Pico Green assay (Quant-IT Pico Green dsDNA assay kit; Invitrogen) on a Tecan Infinite M200Pro (Tecan Group, Ltd.). Prior to all PCRs, all DNA samples were diluted to 4 ng per μl with the elution buffer of the DNA extraction. Primers E341F: 5’-CCTACGGGNGGCWGCAG-3’ and 783Rabc: mixture in 1:1:1 ratio of 5’-CTACCAGGGTATCTAATCCTG-3’; 5’-CTACCGGGGTATCTAATCCCG-3’; 5’-CTACCCGGGTATCTAATCCGG-3’ were used to target the bacterial V3-V4 16S rRNA gene region [18, 19], and primers 5.8SFun: 5’-AACTTTYRRCAAYGGATCWCT-3’ and ITS4fun: 5’-AGCCTCCGCTTATTGATATGCTTAART-3’ were used for the fungal ITS2 region [20]. Reverse primers for 16S V3-V4 region and both the ITS2 primers were selected to reduce co-amplification of plant targets as the soil samples might contain some remaining root fragments. All primers were synthesized (Integrated DNA Technologies, BVBA, Leuven, Belgium) with the universal Illumina MiSeq adapters. Each PCR mixture contained 1x Q5 reaction buffer, 0.2 mM dNTP mix, 0.5 μM each primer, 20 ng template DNA, 1 U Q5 Hot Start High-Fidelity DNA polymerase (New England Biolabs, MA, USA), and nuclease-free water to final volume of 50 μl. The PCR conditions for the target regions 16S V3-V4 and ITS2 were 98 °C for 30 s, followed by resp.15 or 25 cycles of 98 °C for 10 s, resp. 57 or 52 °C for 30 s and 72 °C for 30 s, followed by final elongation at 72 °C for 2 min in a Veriti Thermo Cycler (Thermo Fisher Scientific, USA). Two replicate PCRs were performed in separate runs for each sample for both primer sets, and replicates were pooled per sample and stored at −20 °C. Amplicons were purified, and libraries were prepared according to Illumina guidelines (Illumina, San Diego, CA, USA) and paired-end sequenced with 2×250 cycles for 16S V3-V4 libraries and 2×300 cycles for ITS2 libraries on an Illumina MiSeq platform, and all reads were demultiplexed (Next Generation Sequencing Facilities, Wageningen University & Research, Wageningen, The Netherlands). The 16S rDNA sequences were generated in two sequencing runs to generate more data per sample. The 16S and ITS sequence reads are available at the short read archive (SRA) at NCBI under the accession number PRJNA870764.

The amount of bacterial and fungal DNA was measured by qPCR. For bacteria the primers Eub338: 5’ -ACTCCTACGGGAGGCAGCAG-3’ [21] and Eub518: 5’- ATTACCGCGGCTGCTGG-3’ [22] were used and for fungi the primers ITS1: 5’- TCCGTAGGTGAACCTGCGG-3’ [23] and 5.8S: 5’- CGCTGCGTTCTTCATCG-3’ [24]. Both primer sets were successfully used in qPCR [17] with some remarks: ITS1 is not the reverse but the forward primer and was confusingly named ITS1f [25], as used primer is ITS1 [23] and not ITS1F [25]. TaKaRa TB Green™ Premix Ex Taq II™ (Takara Bio Inc., Japan) was used for the qPCRs. Each reaction contained 1x TaKaRaTB Green Mix, 0.1 μM of each primer, 4 ng of template DNA, and nuclease-free water to a final volume of 10 μl. The qPCR conditions were 95 °C for 2 min, followed by resp. 40 cycles of 95 °C for 10 s and 63 °C for 30 s, followed by the generation of a dissociation curve to verify amplification specificity. Ten-fold serial dilutions of bacterial and fungal DNA (resp. *Mucilaginibacter daejeonensis* and *Fusarium nivale*) ranging from 1000 pg down to 0.01 pg per reaction were included as a calibration curve in triplicate as well as negative controls (no target, nuclease-free water). In addition, the same primers as used for PCR amplicon sequencing were used for conducting qPCRs. However, for the bacterial primer set E341F and 783Rabc, no reliable results were obtained. The results obtained for the fungal primers 5.8SFun and ITS4 were highly correlated with the results from the qPCR primers ITS1 and 5.8S. Therefore, only the results of the latter were used.

### Sequence Data Pre-processing

The pre-processing was done using Qiime2 [26] version 2018.4 on the Galaxy server of the University of Graz in June 2019. The demultiplexed sequence data were denoised using DADA2 [27], which included run-specific quality control and filtering, primer removal, merging of paired-end reads and chimera filtering and generating a feature table with amplicon sequence variants (ASVs) per sample, and a set of unique representative sequences per run as a result. Subsequently, the data of the repeated 16S runs were merged into one data set. Classification was done with a Naïve Bayes classifier [28, 29] using the silva 132 release database [30] for 16S data. The classifier was pretrained on the extracted 16S V3-V4 region of the SILVA database. The ITS sequences were processed as described above. Classification was done with pretrained Naïve Bayes classifier using the Unite developer Qiime release for all Eukaryotes 2019 databases for full-length ITS sequences [31].

### HWC Measurement

Hot water extractable carbon was measured in 2019 as an indicator of soil carbon contained in the soil microbial biomass [32]. It can be used as a cost-effective alternative for more complex measures of soil microbial biomass such as microscopic fungal and bacterial counts. It was assessed as the increase in dissolved carbon after a 16-hour extraction from 4 g soil in 30 ml water at 80 °C as described by Ghani, Dexter, and Perrott [32].

### Statistical Analysis

All statistical analyses were done in RStudio [33].

For assessing differences in disease incidence with and without inoculation with *P. ultimum* and before and after inoculation with *R. solani*, DNA quantity, and HWC, at first, linear mixed models were used with the lmer-function (package lme4) [34], with Block:Land management system as a random variable to account for the split-plot setup of the Soil Health experiment. This model was simplified if factors (soil treatment, land management system, block) had no significant effect, and the random variable was dropped if the explained variance was at least 10 times lower than the variance explained by the main factors. Statistics of mixed models are reported with a *χ*^2^ value and statistics from non-mixed models with an *F*-value. Pairwise differences between treatments were assessed with the emmeans function from the emmeans package [35]. One outlier was removed from the fungal quantification data having an approximately 10-fold higher value than the remaining data.

The sequence data were further analyzed using the package phyloseq [36]. OTUs occurring less than 20 times in the whole dataset were removed. In addition, OTUs were filtered with a prevalence threshold of 4 (number of samples the OTU had to occur in). The Shannon diversity index and differences in diversity between land management systems and treatments were determined for each sample within the microeco package [37]. Multivariate analyses were carried out with nonmetric multidimensional scaling (NMDS). Significant differences in microbial community composition between land management systems and treatments were assessed with permanova analysis using the adonis function from the vegan package [38]. Differential abundance analysis of bacterial and fungal taxa between land management systems and treatments was determined with the package DESeq2 [39]. In addition, the package randomForest was used to assess which taxa are associated with the disease incidence in the presence of *P. ultimum* [40]. This was done on the sequence dataset from all treatments filtered for abundance with a threshold of 0.5 relative abundance for bacteria and 0.1 for fungi. This difference was due to the high amount of OTUs in the bacterial dataset making a more stringent filtering necessary. The main difference between the differential abundance analysis and the randomForest prediction is that differential abundance analysis considers all taxa separately, while randomForest assesses which combination of taxa best predicts the outcome (here disease incidence as a continuous variable).

## Results

### Disease Suppressiveness (2018)

#### ***P. ultimum Bioassay***

**Fig. 2:**
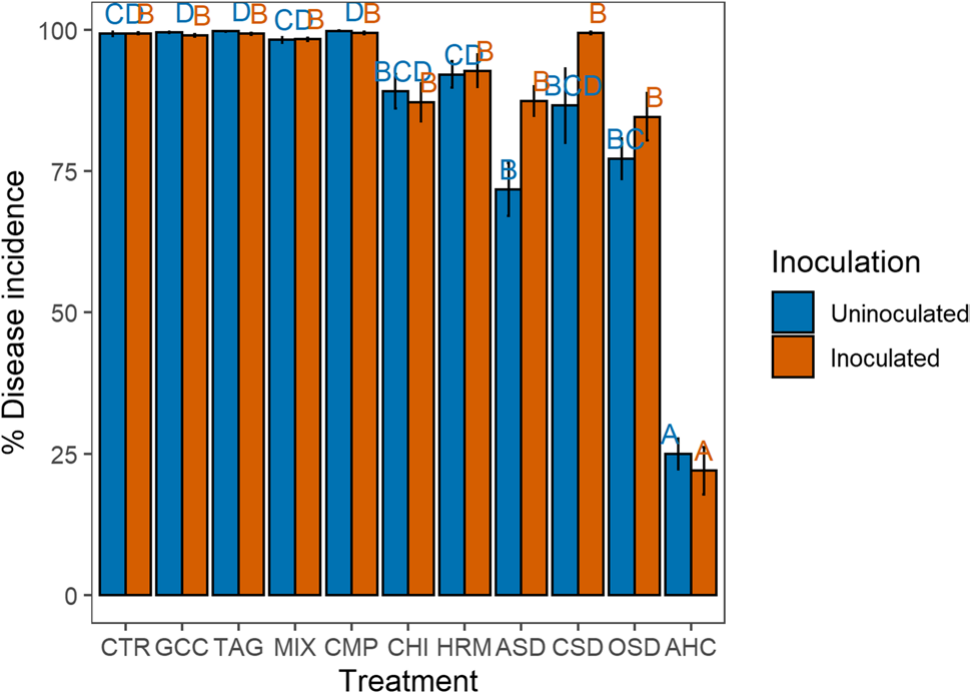
Disease incidence with and without inoculation with *P. ultimum* in 2018 in the 11 treatments averaged over land management (*n*=8 and *n*=4 for OSD and CSD). The error bars represent the standard error; different letters indicate significant differences between the treatments (*p*<0.05)

Natural infection with *P. ultimum* was on average 86% but differed significantly between treatments (*χ*^2^=20.29, *p*<0.01), though not between land management systems. Natural infection was lowest in the AHC treatment followed by the ASD and OSD treatments (Fig. 2). When *P. ultimum* was added, only the AHC treatment showed a significantly lower disease incidence compared to all other treatments (*χ*^2^=10.59, *p*=0.01) (Fig. 2). Also, for disease incidence with the addition of *P. ultimum*, there was no difference between land management systems (*χ*^2^=0.35, *p*=0.56).

In addition, the fresh weight of the above-ground plant parts with the addition of *P. ultimum* differed significantly between treatments (*χ*^2^=97.98, *p*<0.01) and was highest in the AHC treatment (Fig. S1).

#### ***R. solani Bioassay***

**Fig. 3:**
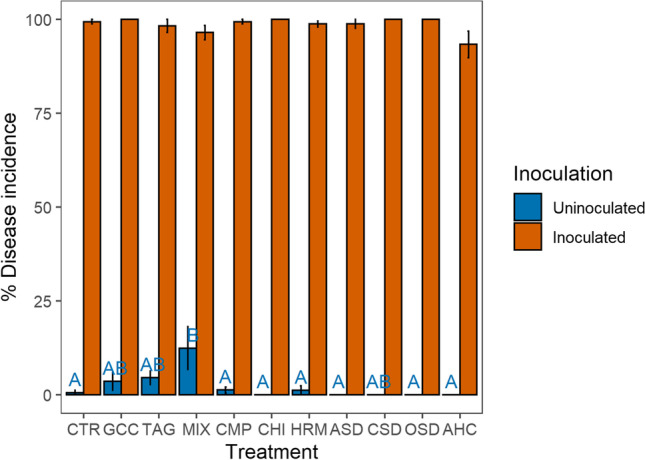
Disease incidence before and after inoculation with *R. solani* in 2018 in the 11 treatments averaged over land management (*n*=8 and *n*=4 for OSD and CSD). The error bars represent the standard error; different letters indicate significant differences between the treatments (*p*<0.05)

Natural infection with *R. solani*, measured as disease incidence without inoculation, was low (on average 2%) but significantly higher in the organic land management system (3.6%) compared to the conventional system (1.1%) (*χ*^2^=24.17, *p*=0.04) (Fig. S2). Natural infection also differed between treatments (*χ*^2^=32.70, *p*<0.01) with infection being highest in the MIX treatment and lowest in the CHI, ASD, OSD, and AHC treatments (Fig. 3). After inoculation with *R. solani*, total average disease incidence was 98%, and no significant treatment and management effects could be detected.

### Disease Suppressiveness (2019)

#### ***P. ultimum Bioassay***

**Fig. 4:**
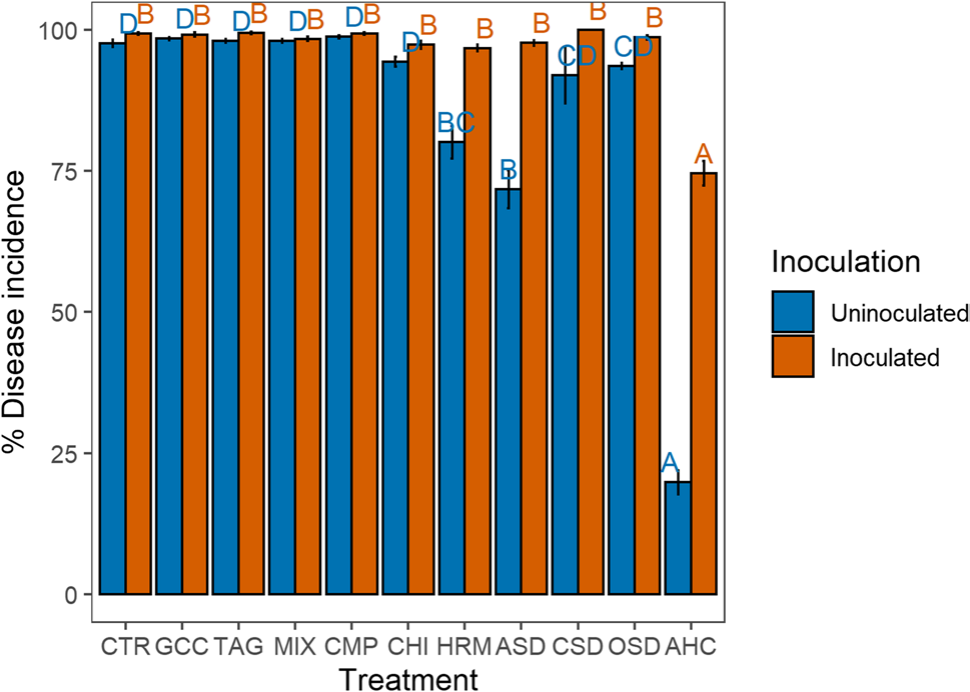
Disease incidence with and without inoculation with *P. ultimum* in 2019 in the 11 treatments averaged over land management (*n*=8 and *n*=4 for OSD and CSD). The error bars represent the standard error; different letters indicate significant differences between the treatments (*p*<0.05)

Natural infection in the bioassay with *P. ultimum* in 2019 was on average 85% and differed between the treatments (*χ*^2^=792, *p*<0.01) (Fig. 4). Disease incidence was significantly lower in the AHC treatment, followed by the ASD and HRM treatments. Inoculation with *P. ultimum* led to differences in disease incidence between the treatments (*χ*^2^=521.48, *p*<0.01), with only the AHC treatment showing a lower disease incidence than the other treatments (Fig. 4). There were no differences between the organic and conventional system (natural infection: *χ*^2^=1.88, p=0.17, disease incidence after inoculation: *χ*^2^=1.41, *p*=0.23).

In addition, the fresh weight of the above-ground plant parts with the addition of *P. ultimum* differed between the treatments (*χ*^2^=184.80, *p*<0.01) being highest in the AHC treatment (Fig. S3).

#### ***R. solani Bioassay***

**Fig. 5:**
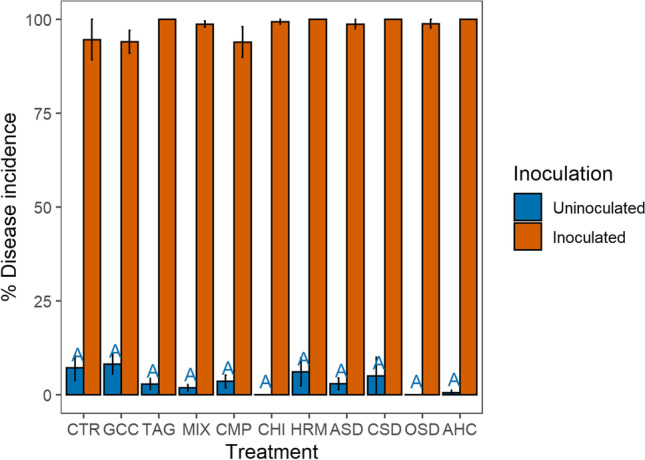
Disease incidence before and after inoculation with *R. solani* in 2019 in the 11 treatments averaged over land management (n=8 and n=4 for OSD and CSD); the error bars represent the standard error; different letters indicate significant differences between the treatments (p<0.05)

Natural infection with *R. solani* did not differ significantly between land management systems (*χ*^2^=2.57, *p*=0.11) but was significantly higher in the CTR and GRK treatment compared to the CHI and AHC treatment (*χ*^2^=18.18, *p*=0.05) (Fig. 5). There was no difference in total disease incidence after inoculation (land management: *χ*^2^=0.34, *p*=0.56, treatment: *χ*^2^ =12.25, *p*=0.27).

### Microbiome Composition (2018)

#### 16S rRNA

**Fig. 6:**
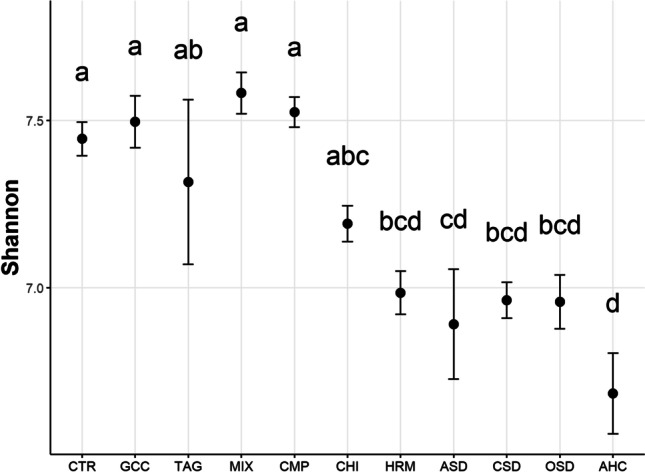
Shannon diversity of bacteria in the 11 treatments. Different letters indicate significant differences between the treatments (*p*<0.05)

After filtering, 14652 OTUs were retained. Bacterial alpha diversity did not differ between conventional and organic management but differed between treatments. The AHC treatment had the lowest diversity index followed by the ASD, CSD, OSD, and HRM treatments (Fig. 6). The highest diversity was found in the CTR, MIX, CMP, and GCC treatments. Also, observed diversity (i.e., OTU richness) was lowest in the AHC, ASD, CSD, OSD, and HRM treatments (Fig. S4).

**Fig. 7:**
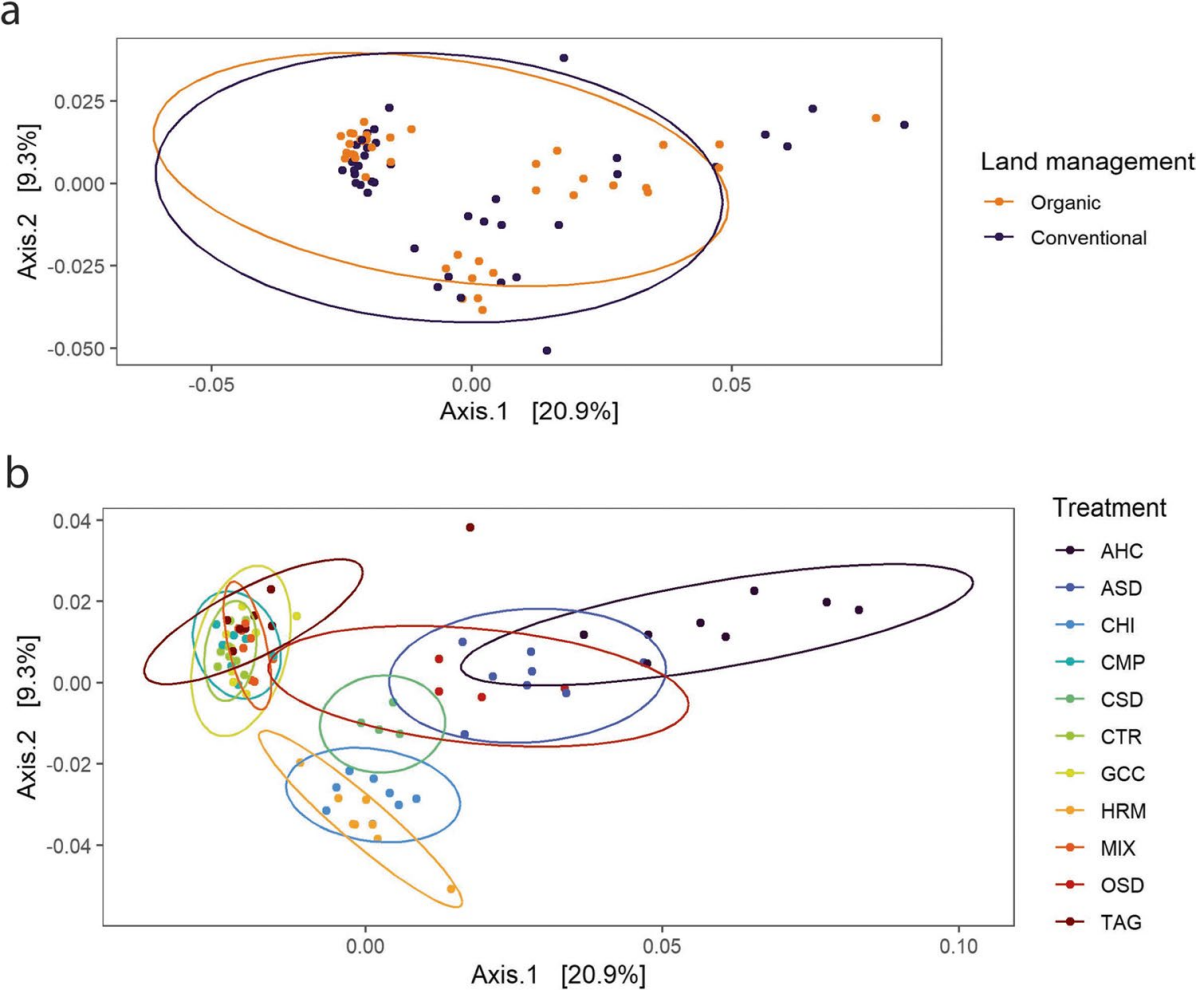
NMDS analysis of bacterial communities in all samples. Each dot represents a sample; samples are colored by **a** land management and **b** treatment

A NMDS analysis of all samples showed a high similarity between bacterial communities from organic and conventional fields (Fig. 7a). Nevertheless, differences between the two land management systems were significant as assessed by permanova adjusting for block effects (*F*-model=3.71, *p*<0.01). Communities clustered visibly by treatment (Fig. 7b) (*F*-model=6.11, *p*<0.01), with the treatments CTR, GCC, TAG, CMP, and MIX clustered together. The HRM treatment overlapped with the CHI treatment, and the AHC treatment partly overlapped with the ASD and OSD treatments, while the CSD treatment showed an overlap with both clusters.

Sixty-nine taxa grouped into 28 genera differed significantly in abundance between conventional and organic land management (Fig. S5a). A total of 1397 taxa differed in abundance between the CTR and AHC treatment, of which 686 taxa, grouped into 34 genera, were significantly increased in abundance in the AHC treatments. These taxa included *Stenotrophomonas* sp., *Pseudomonas* sp., and *Flavobacterium* sp. (Fig. S5b).

**Fig. 8:**
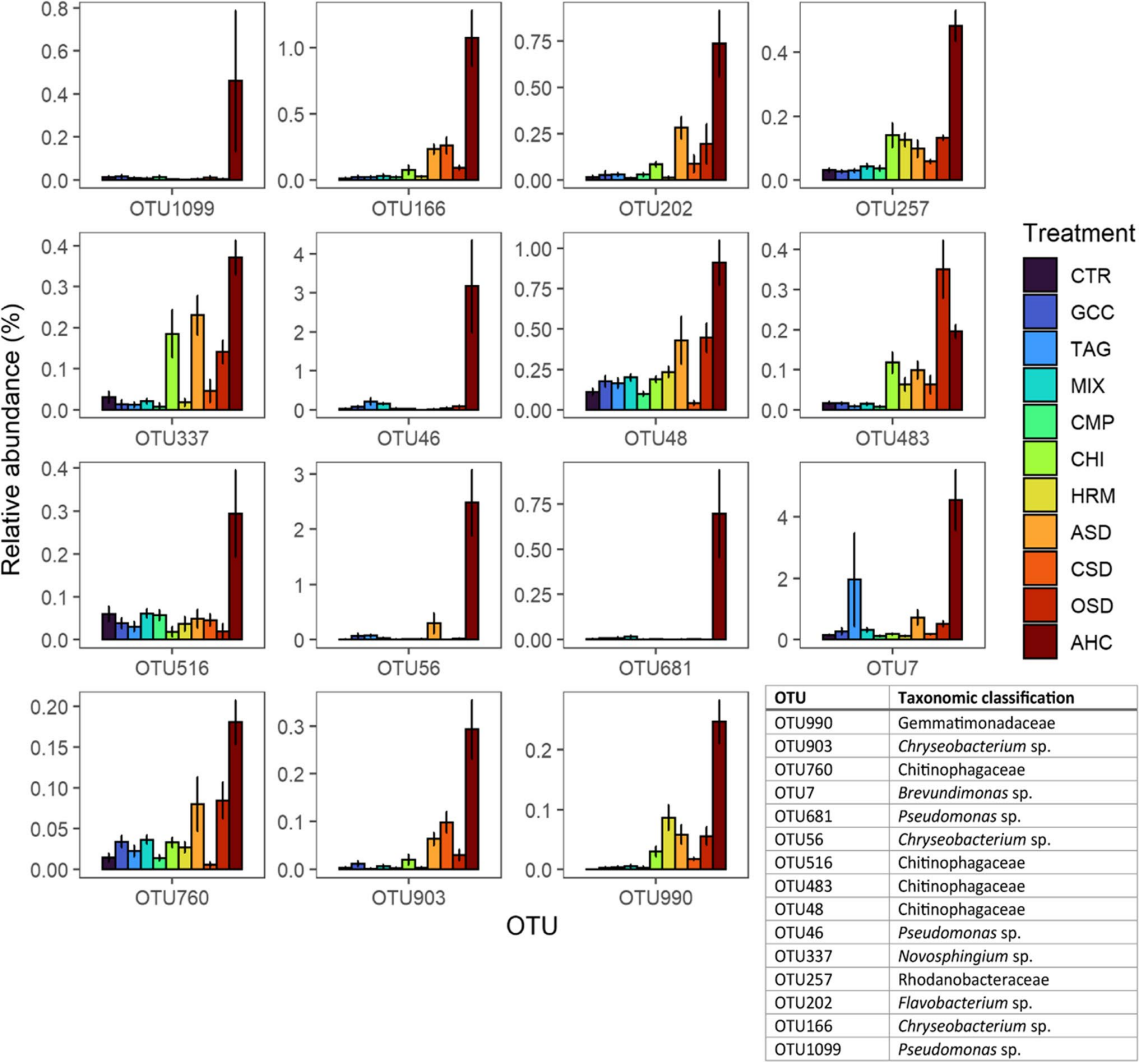
Average relative abundance of 15 OTUs, which were the most important statistical predictors for differences between samples disease incidence with *P. ultimum* in the 11 treatments averaged over management system. The error bars represent the standard error

A machine learning approach was used to assess which taxa can be used to predict disease incidence after inoculation with *P. ultimum*. The results of this approach explained 71% of the variation in disease incidence. The 15 OTUs that were most important in predicting disease incidence were in most cases more abundant in the AHC treatment compared to the other treatments and therefore predictive of a comparatively lower disease incidence (Fig. 8). Notably, several belonged to the family Chitinophagaceae and the genera *Chryseobacterium* and *Pseudomonas*.

#### ITS

**Fig. 9:**
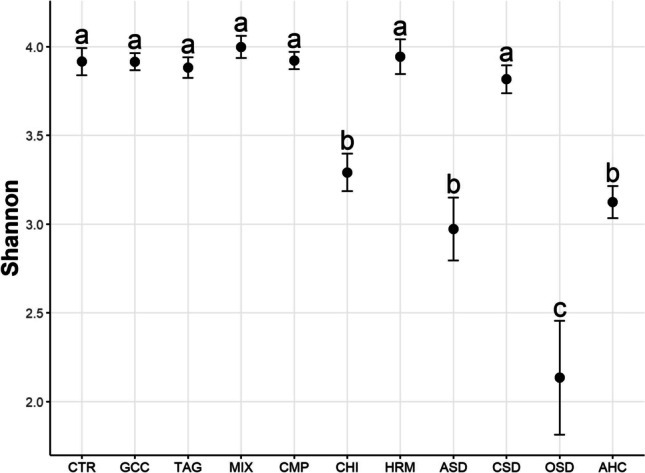
Shannon diversity of fungi in the 11 different treatments. Different letters indicate significant differences between the treatments (*p*<0.05)

After filtering, 1402 fungal OTUs were retained in the dataset. There was no difference in Shannon diversity between the two land management systems. However, there were differences between treatments (Fig. 9). The lowest alpha diversity was found in the OSD treatment, followed by the AHC, ASD, and CHI treatments. The other treatments did not significantly differ from each other. Also observed diversity was lower in the AHC, ASD, CHI, and OSD treatments (Fig. S6). It was also significantly lower in the CSD treatment, although Shannon diversity did not differ from the control.

**Fig. 10:**
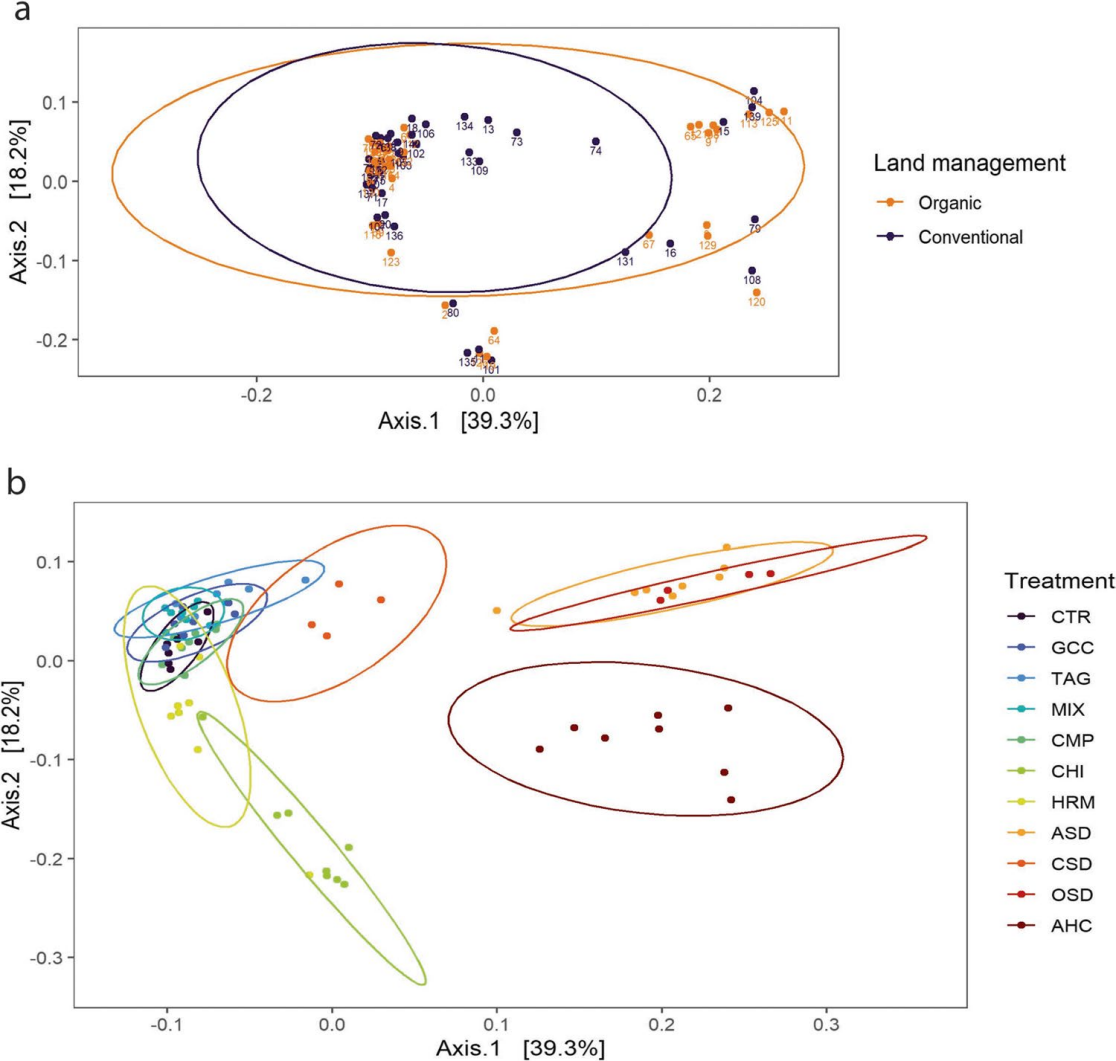
NMDS analysis of fungal communities in all samples. Each dot represents a sample; samples are colored by **a** land management and **b** treatment

A NMDS analysis showed a large overlap between fungal communities from conventional and organic land management (Fig. 10a). Still, a permutation test showed significant differences (*F*-model=6.27, *p*<0.01). Also, treatments differed significantly from each other (*F*-model=15.11, *p*<0.01). Especially, the AHC treatment clustered separately (Fig. 10b). Another cluster was formed by the ASD and OSD treatments. Also, the CHI ant CSD treatments showed only limited overlap with the cluster of the remaining treatments.

Ninety-one taxa, belonging to 20 genera, significantly differed in abundance between the land management systems (Fig. S7a). A total of 203 taxa differed in abundance between the CTR and AHC treatment, of which 112 were more abundant in the AHC treatment belonging to 17 genera (Fig. S7b).

**Fig. 11:**
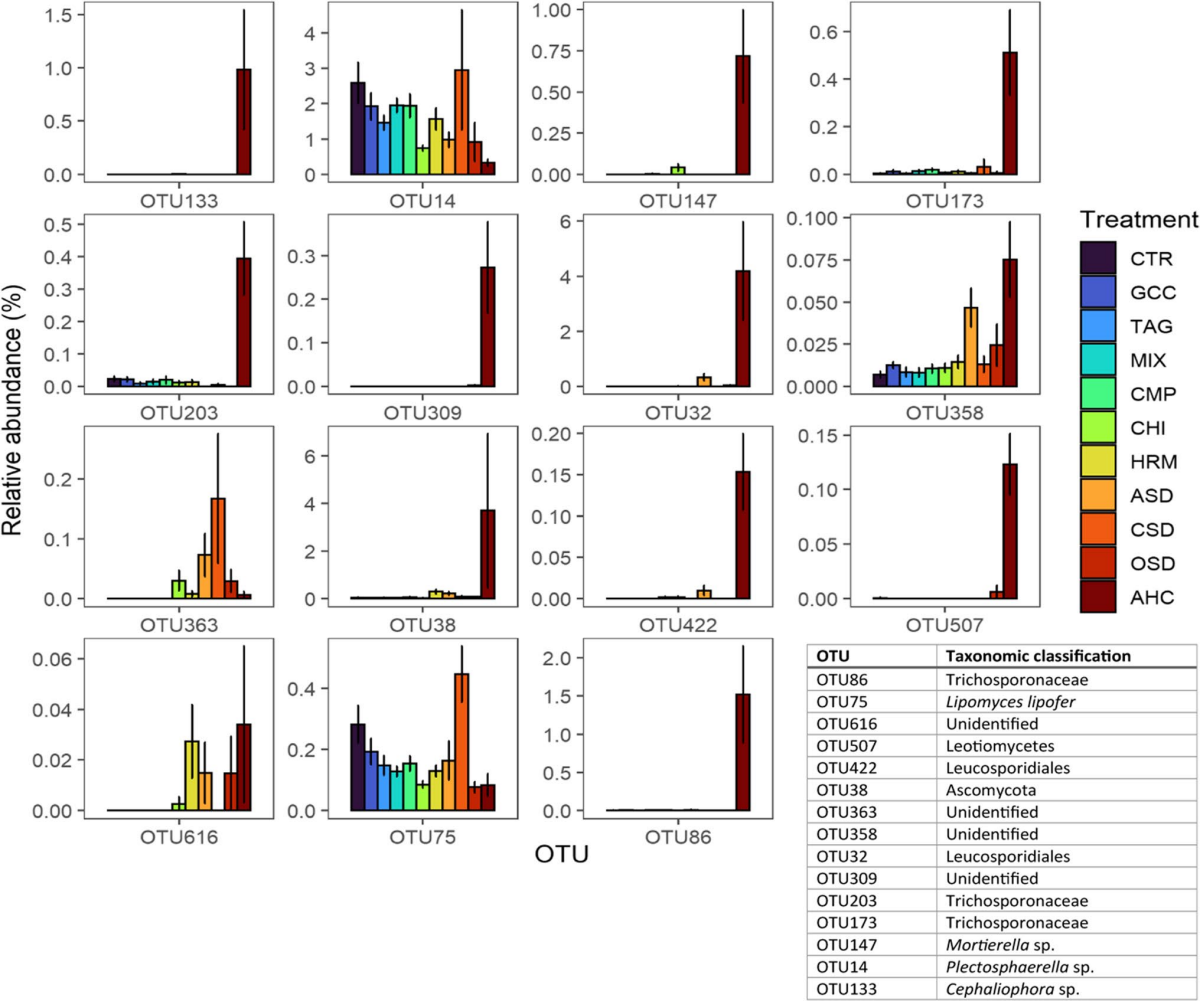
Average relative abundance of 15 OTUs, which were the most important statistical predictors for differences between samples in disease incidence with *P. ultimum* in the 11 treatments averaged over land management system. The error bars represent the standard error

A machine learning approach explained 65% of the variance in *P. ultimum* suppressiveness. Among the 15 taxa that were most important in statistically predicting disease incidence with *P. ultimum* were several strains of the family Trichosporonaceae and the order Leucosporidiales, which were most abundant in the AHC treatment (Fig. 11).

### Microbial Quantification (2018)

The amount of fungal and bacterial DNA was assessed by qPCR. Both fungal and bacterial DNA measurements did not differ significantly between the land management systems but differed between treatments (*χ*^2^=117.48, p<0.01 and *χ*^2^=57.60, *p*<0.01 respectively). The amount of bacterial DNA was highest in the AHC. The other treatments did not differ significantly from each other (Fig. S8). The amount of fungal DNA was highest in the OSD treatments followed by the AHC and the ASD and CHI treatments (Fig. S9), but lowest in the CSD treatment.

### Hot Water Extractable Carbon (HWC) (2019)

**Fig. 12:**
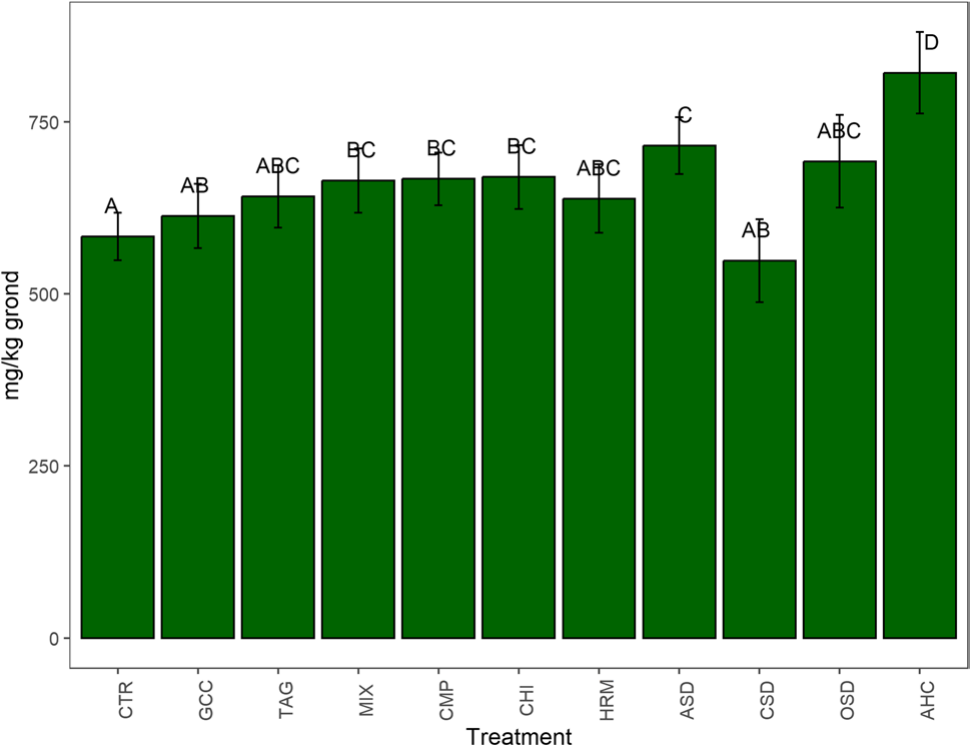
Hot water extractable carbon (HWC) in 2019 in the 11 different treatments averaged over land management. The error bars represent the standard error; different letters indicate significant differences between the treatments (*p*<0.05)

HWC did not differ significantly between the land management system, but differed between treatments (*χ*^2^=139.25, *p*<0.01). HWC was highest in the AHC treatment followed by the ASD treatment (Fig. 12). Also, the treatments MIX, CMP, and CHI contained significantly more HWC than CTR.

### Crop Yield (2019)

The crop yield of leek in 2019 is reported in Table S3. Yield did not differ significantly between treatments, but between the two management systems (*χ*^2^=6.68, *p*=0.01). A higher average yield of 45.4 was obtained from the conventional system compared to 41.21 from the organic system.

## Discussion

In this study, we compared 11 soil treatments in combination with organic and conventional land management regarding their effect on the soil microbial community and disease suppressiveness, indicated by a relatively lower disease incidence after inoculation with the pathogen compared to the control. In summary, we found that for *R. solani* AG 2-2IIIB only natural infection but not disease suppressiveness was affected by the treatments. In contrast, suppressiveness against *P. ultimum* was increased in the AHC treatment that combined anaerobic soil disinfestation, hair meal, and compost amendment. For none of the pathogens, an effect of land management on disease suppressiveness could be detected.

Natural infection with *R. solani* was increased in the organic land management system in 2018 and in both years in the mixed cover crop treatments (MIX). *R. solani* might have multiplied in host plants among the cover crops, such as the potential host *Lolium multiflorum* (Italian ryegrass) or on the incorporated organic material [41, 42]. In contrast, the soil health treatments chitin and AHC showed low natural infection in both years. Chitin application, which led to almost no detectable infection, has been shown to effectively reduce fungal pathogens before, likely by increasing the abundance of chitinolytic bacteria [10]. Also, disinfestation treatments were proven to be effective against *R. solani* [43]. On average, natural infection was low and highly variable in the soil health experiment, which might have prevented the detection of strong treatment and management effects. Nevertheless, after inoculation with *R. solani* in the bioassays, disease incidence was high in all treatments, indicating no suppressiveness. Disease suppressiveness against this particular pathogen is believed to be mediated by the action of specific bacterial and fungal taxa, such as members of the Oxalobacteraceae, Bacillaceae, and Mortierellaceae [10, 44]. In this study, several taxa that are expected to be effective against *R. solani*, such as *Pseudomonas* sp., *Massilia* sp., and *Mortierella* sp. [10, 45], were increased in abundance in the AHC treatment. However, no suppressiveness could be detected. This might be due to microbial genera and even species containing a number of strains with very different traits and functions. Since most studies, including this one, cannot identify taxa to strain level, the taxa that were enhanced could be very different from those reported earlier for disease suppression. Alternatively, the setup of the bioassay, e.g., type and amount of *R. solani* and inoculum, could have prevented us from detecting suppressiveness.

Natural infection with *Pythium ultimum* was high in almost all plots. The same was observed by Bongiorno et al. [16] in an experimental field at the same location as in the present study. In general, increased infections with *Pythium ultimum* have been observed on sandy soils in the Netherlands in recent years (personal communication Joeke Postma). Potentially, fluctuations in soil moisture due to more extreme weather conditions favored the establishment of the pathogen. Still, natural infection with *P. ultimum* was reduced in treatments containing anaerobic soil disinfestation, indicating a detrimental effect on the pathogen. It has been reported that volatile organic compounds (VOCs) released during disinfestation were responsible for the reduction of fungal pathogens [46]. In addition, in the present study, we also found a relatively lower disease incidence after inoculation *P. ultimum* when anaerobic soil disinfestation was followed by the addition of two types of organic material (hair meal and compost), which indicates suppressiveness of the soil. *Pythium* suppressiveness has in several studies been associated with general suppressiveness, thus an increase in microbial biomass and activity and therefore competition with the pathogen [47]. In accordance with these findings, we found a higher bacterial DNA content in the AHC treatment and an increase in HWC which is a measure of the carbon from microbial community. Possibly the eradication of a part of the microbial community by disinfestation followed by organic amendments led to a rapid increase of fast-growing microorganisms resulting in general suppressiveness. Compost application is known to stimulate microbial biomass and activity and thus has been frequently found to increase soil suppressiveness [48]. Also, fungal DNA was increased in the AHC treatment, but was even higher in the OSD treatment. However, there was a high variation in the OSD treatment, and it is even possible that the selected assay for fungal quantification also detected plant DNA.

It could also be that disease suppressiveness was caused by a shift in the microbial community composition by first reducing competition and then supplying substrates favorable to certain species. This is supported by the finding that both the bacterial and the fungal community composition differed markedly in the AHC treatment from the other treatments. While previous studies found that microorganisms introduced with compost can be responsible for suppressiveness [45, 49], the taxa found to be related to suppressiveness in the present study were also present in treatments not receiving compost. This suggests that those taxa might have originated from soil and were selectively stimulated by the treatment. Compost application has for example been demonstrated to stimulate Actinomycetes, which are associated with suppressiveness against fungi [50]. Differential abundance analysis in the present study revealed a relatively higher abundance of the Actinomycete *Streptomyces* sp. in the AHC treatment, a genus which is known for its plant growth-promoting ability and suppressive activity against a number of soil-borne diseases [51, 52]. The addition of hair meal could have led to an increase in the relative abundance of keratinolytic bacteria [53]. Among the taxa that were increased in the AHC treatment were genera like *Stenotrophomonas* and *Chryseobacterium* which are known to be potentially keratinolytic. *Chryseobacterium* has further been found to show antagonistic activity against *P. ultimum* [54, 55]. Also, various *Pseudomonas* species are known to produce a range of antibacterial and antifungal compounds, but it cannot be excluded that also other mechanisms such as siderophore production and competition for nutrients play a role in suppressiveness [56]. In addition, the abundance of members of the bacterial family Chitinophagaceae was a statistical predictor of *Pythium* suppressiveness. This particular family has frequently been associated with the control of fungal pathogens [57], but its effect against *Pythium* has not been investigated yet. In the present study also a number of fungal taxa were associated with *Pythium* suppressiveness. The fungal genus *Mortierella* was statistically predictive of disease suppressiveness against *P. ultimum*. It was shown previously that suppressive soils against *Fusarium* wilt disease contained higher abundances of this genus, although the mechanism behind is not yet known [58, 59]. Members of the fungal family Trichosporonaceae were also more abundant in the AHC treatment and correlated with disease suppressiveness. This family contains highly diverse genera and species. However, soil-borne species were found to be able to degrade complex substrates [60], which might explain their increased abundance in a treatment with compost as well as hair meal addition. This family has not yet been described as being associated with disease suppressiveness.

Interestingly, the diversity indices for both bacteria and fungi were decreased in the AHC treatment compared to the control. This is in contrast to the assumption that the addition of organic material would increase diversity [61]. In addition, it is frequently assumed that a lower diversity would lead to a decreased suppressiveness as less diverse communities would be more prone to the invasion of pathogens due to a less complementary use of resources [5]. However, disease suppressiveness was strongest in the AHC treatment. This can be explained by the addition of hair meal and compost amendments, which has likely led to an increase in a subset of species, which were effective in *Pythium* suppressiveness. A similar effect has been found by Andreo-Jimenez et al. [10], who showed that keratin addition decreased the bacterial diversity, but increased suppressiveness against *R. solani*, likely by leading to an increase in the abundance of bacterial taxa like the genus *Flavobacterium*. Still, diversity is essential for this increase in suppressiveness. Only a sufficient diversity in the untreated soil can ensure that the groups necessary for suppressiveness are present and able to multiply under favorable conditions.

In contrast to our expectations, there was only a little difference in microbial community composition between the organic and the conventional land management system. While some bacterial and fungal species differed in abundance between the two, there were no differences in HWC or disease suppressiveness. It should be noted that the two management systems in this study were kept as similar as possible with regard to crop rotation and nutrient input. Neither did tillage practices differ. Both management systems can be seen as highly intensive arable systems with high crop yields

Microbial community composition was only assessed in 2018, and therefore, it cannot be determined if the applied treatments had a long-term effect on the soil microbiome. However, it is likely that the changes detected in 2018 persist for at least 1 year in the AHC treatment since there was still suppressiveness against *P. ultimum* 1 year after the treatments. This indicates that reduction by disinfestation followed by stimulation by using organic amendments can induce lasting change in the microbial community composition. In previous studies, there was no evidence that anaerobic disinfestation or compost amendment separately changed microbial community composition for more than a year [46, 62], but we could not find reports on a combination of treatments. Ideally, microbial community composition should be monitored in regular intervals after the application of the treatments and over longer periods to assess if the changes are permanent or transient. This is especially important for farmers, since some treatments can be costly.

## Conclusion

By comparing eleven different soil health treatments and two land management systems in a common field, we could show that a combination treatment of anaerobic soil disinfestation, hair meal, and compost amendment increased fungal and bacterial biomass, altered the microbiome, and increased suppressiveness against *P. ultimum*. This effect could still be detected 1 year after the application of the treatment. On the other hand, there were no differences in suppressiveness between conventional and organic land management.

This study indicates that while the effect of single soil treatments is often limited, a combination can lead to substantial changes in the microbial community composition and to persistent effects on disease suppressiveness. Future studies will have to show if there is a causal relationship between the microbial taxa identified in this study and suppressiveness. Moreover, here, we only detected suppressiveness against *P. ultimum*. It can be assumed that different combinations of reductive treatments and soil amendments will lead to different changes in the soil microbiome and thereby potentially to suppressiveness against other pathogen species. More combinations of soil health treatments need to be tested to determine their potential in increasing soil suppressiveness.

## **Supplementary Information**

ESM 1

## Data Availability

The datasets generated during and/or analyzed during the current study are available from the corresponding author on reasonable request.

## References

[1] Hakim S, Naqqash T, Nawaz MS, Laraib I, Siddique MJ, Zia R, Mirza MS, Imran A (2021). Rhizosphere engineering with plant growth-promoting microorganisms for agriculture and ecological sustainability. Front Sustain Food Syst..

[2] Weller DM, Raaijmakers JM, Gardener BBM, Thomashow LS (2002). Microbial populations responsible for specific soil suppressiveness to plant pathogens. Annu Rev Phytopathol..

[3] Schlatter D, Kinkel L, Thomashow L, Weller D, Paulitz T (2017). Disease suppressive soils: new insights from the soil microbiome. Phytopathology..

[4] Yadav R, Panwar J, Meena H, Thirumalaisamy P, Meena R (2015). Developing disease-suppressive soil through agronomic management, organic amendments and soil suppressiveness in plant disease management.

[5] van Elsas JD, Garbeva P, Salles J (2002). Effects of agronomical measures on the microbial diversity of soils as related to the suppression of soil-borne plant pathogens. Biodegradation..

[6] Mazzola M (2002). Mechanisms of natural soil suppressiveness to soilborne diseases. Antonie van Leeuwenhoek..

[7] Letourneau D, van Bruggen A (2006). Crop protection in organic agriculture.

[8] Hiddink GA, van Bruggen AHC, Termorshuizen AJ, Raaijmakers JM, Semenov AV (2005). Effect of organic management of soils on suppressiveness to *Gaeumannomyces graminis* var. *tritici* and its antagonist, *Pseudomonas fluorescens*. Eur J Plant Pathol..

[9] De Corato U (2020). Disease-suppressive compost enhances natural soil suppressiveness against soil-borne plant pathogens: a critical review. Rhizosphere..

[10] Andreo-Jimenez B, Schilder MT, Nijhuis EH, DEt B, Bloem J, JHM V, Gv O, Brolsma K, Wd B, Postma J, Stabb EV (2021). Chitin- and keratin-rich soil amendments suppress *Rhizoctonia solani* disease via changes to the soil microbial community. Appl Environ Microbiol..

[11] Bonanomi G, Lorito M, Vinale F, Woo SL (2018). Organic amendments, beneficial microbes, and soil microbiota: toward a unified framework for disease suppression. Annu Rev Phytopathol..

[12] Panth M, Baysal-Gurel F, Simmons T, Addesso KM, Witcher A (2020). Impact of winter cover crop usage in soilborne disease suppressiveness in woody ornamental production system. Agronomy..

[13] Wen L, Lee-Marzano S, Ortiz-Ribbing LM, Gruver J, Hartman GL, Eastburn D (2017). Suppression of soilborne diseases of soybean with cover crops. Plant Dis..

[14] Lupatini M, Korthals GW, de Hollander M, Janssens TK, Kuramae EE (2017). Soil microbiome is more heterogeneous in organic than in conventional farming system. Front Microbiol..

[15] Uzuhashi S, Kakishima M, Tojo M (2010). Phylogeny of the genus Pythium and description of new genera. Mycoscience..

[16] Bongiorno G, Postma J, Bünemann EK, Brussaard L, de Goede RG, Mäder P, Tamm L, Thuerig B (2019). Soil suppressiveness to *Pythium ultimum* in ten European long-term field experiments and its relation with soil parameters. Soil Biol Biochem..

[17] Postma J, Schilder MT, Bloem J, van Leeuwen-Haagsma WK (2008). Soil suppressiveness and functional diversity of the soil microflora in organic farming systems. Soil Biol Biochem..

[18] Herlemann DPR, Labrenz M, Jürgens K, Bertilsson S, Waniek JJ, Andersson AF (2011). Transitions in bacterial communities along the 2000 km salinity gradient of the Baltic Sea. ISME J..

[19] Sakai M, Matsuka A, Komura T, Kanazawa S (2004). Application of a new PCR primer for terminal restriction fragment length polymorphism analysis of the bacterial communities in plant roots. J Microbiol Methods..

[20] Taylor DL, Walters William A, Lennon Niall J, Bochicchio J, Krohn A, Caporaso JG, Pennanen T, Cullen D (2016). Accurate estimation of fungal diversity and abundance through improved lineage-specific primers optimized for Illumina amplicon sequencing. Appl Environ Microbiol..

[21] Lane D (1991). 16S/23S rRNA sequencing. Nucleic acid techniques in bacterial systematics.

[22] Muyzer G, De Waal EC, Uitterlinden A (1993). Profiling of complex microbial populations by denaturing gradient gel electrophoresis analysis of polymerase chain reaction-amplified genes coding for 16S rRNA. Appl Environ Microbiol..

[23] White TJ, Bruns T, Lee S, Taylor J (1990). Amplification and direct sequencing of fungal ribosomal RNA genes for phylogenetics. PCR protocols: a guide to methods and applications.

[24] Vilgalys R, Hester M (1990). Rapid genetic identification and mapping of enzymatically amplified ribosomal DNA from several *Cryptococcus* species. J Bacteriol..

[25] Gardes M, Bruns TD (1993). ITS primers with enhanced specificity for basidiomycetes-application to the identification of mycorrhizae and rusts. Mol Ecol..

[26] Bolyen E, Rideout JR, Dillon MR, Bokulich NA, Abnet CC, Al-Ghalith GA, Alexander H, Alm EJ, Arumugam M, Asnicar F, Bai Y (2019). Reproducible, interactive, scalable and extensible microbiome data science using QIIME 2. Nat Biotechnol..

[27] Callahan B, McMurdie P, Rosen M, Han A, Johnson A, Holmes S (2016). DADA2: High-resolution sample inference from Illumina amplicon data. Nat Methods..

[28] Pedregosa F, Varoquaux G, Gramfort A, Michel V, Thirion B, Grisel O, Blondel M, Prettenhofer P, Weiss R, Dubourg V (2011). Scikit-learn: machine learning in Python. J Mach Learn Res..

[29] Bokulich NA, Kaehler BD, Rideout JR, Dillon M, Bolyen E, Knight R, Huttley GA, Gregory Caporaso J (2018). Optimizing taxonomic classification of marker-gene amplicon sequences with QIIME 2’s q2-feature-classifier plugin. Microbiome..

[30] Quast C, Pruesse E, Yilmaz P, Gerken J, Schweer T, Yarza P, Peplies J, Glöckner FO (2013). The SILVA ribosomal RNA gene database project: improved data processing and web-based tools. Nucleic Acids Res..

[31] Community U (2019). UNITE QIIME release for Fungi.

[32] Ghani A, Dexter M, Perrott K (2003). Hot-water extractable carbon in soils: a sensitive measurement for determining impacts of fertilisation, grazing and cultivation. Soil Biol Biochem..

[33] RStudio T (2016). RStudio: Integrated Development Environment for R.

[34] Bates D, Mächler M, Bolker B, Walker S (2015). Fitting linear mixed-effects models using (lme4). J Stat Softw..

[35] Lenth R, Singmann H, Love J, Buerkner P, Herve M (2018). emmeans: estimated marginal means, aka least-square means.

[36] McMurdie PJ, Holmes S (2013). phyloseq: An R package for reproducible interactive analysis and graphics of microbiome census data. PLOS ONE.

[37] Liu C, Cui Y, Li X, Yao M (2020). microeco: an R package for data mining in microbial community ecology. FEMS Microbiol Lett..

[38] Oksanen J, Blanchet FG, Kindt R, Legendre P, Minchin PR, O'hara RB, Oksanen M (2013). Package ‘vegan’. Community ecology package, version.

[39] Love MI, Huber W, Anders S (2014). Moderated estimation of fold change and dispersion for RNA-seq data with DESeq2. Genome Biol..

[40] Liaw A, Wiener M (2002). Classification and regression by randomForest. R News..

[41] Neate SM (1987). Plant debris in soil as a source of inoculum of Rhizoctonia in wheat. Trans Br Mycol Soc..

[42] Westerdijk CE, Schneider JHM (2001). Rhizoctonia solani in suikerbieten : inzet groenbemesters beperkt schade. PPO-bulletin akkerbouw..

[43] Rosskopf E, Di Gioia F, Hong JC, Pisani C, Kokalis-Burelle N (2016). Organic amendments for pathogen and nematode control. Annu Rev Phytopathol..

[44] Liu X, Hannula SE, Li X, Hundscheid MPJ, Klein Gunnewiek PJA, Clocchiatti A, Ding W, de Boer W (2021). Decomposing cover crops modify root-associated microbiome composition and disease tolerance of cash crop seedlings. Soil Biol Biochem..

[45] Pane C, Spaccini R, Piccolo A, Scala F, Bonanomi G (2011). Compost amendments enhance peat suppressiveness to *Pythium ultimum*, *Rhizoctonia solani* and *Sclerotinia minor*. Biol Control..

[46] van Agtmaal M, van Os G, Hol G, Hundscheid M, Runia W, Hordijk C, De Boer W (2015). Legacy effects of anaerobic soil disinfestation on soil bacterial community composition and production of pathogen-suppressing volatiles. Front Microbiol..

[47] Van Os G, Van Ginkel J (2001). Suppression of Pythium root rot in bulbous Iris in relation to biomass and activity of the soil microflora. Soil Biol Biochem..

[48] D’Hose T, Ruysschaert G, Viaene N, Debode J, Vanden Nest T, Van Vaerenbergh J, Cornelis W, Willekens K, Vandecasteele B (2016). Farm compost amendment and non-inversion tillage improve soil quality without increasing the risk for N and P leaching. Agric Ecosyst Environ..

[49] Mayerhofer J, Thuerig B, Oberhaensli T, Enderle E, Lutz S, Ahrens CH, Fuchs JG, Widmer F (2021). Indicative bacterial communities and taxa of disease-suppressing and growth-promoting composts and their associations to the rhizoplane. FEMS Microbiol Lett..

[50] van Elsas JD, Postma J, Diaz LF, de Bertoldi M, Bidlingmaier W, Stentiford E (2007). Chapter 10 Suppression of soil-borne phytopathogens by compost. Waste Management Series.

[51] Whipps JM, Lumsden RD (1991). Biological control of Pythium species. Biocontrol Sci Technol..

[52] Viaene T, Langendries S, Beirinckx S, Maes M, Goormachtig S (2016) Streptomyces as a plant's best friend? FEMS Microbiol Lett. 92. 10.1093/femsec/fiw119 10.1093/femsec/fiw119 27279415

[53] Tamreihao K, Mukherjee S, Khunjamayum R, Devi LJ, Asem RS, Ningthoujam DS (2019). Feather degradation by keratinolytic bacteria and biofertilizing potential for sustainable agricultural production. J Basic Microbiol..

[54] Dunne C, Crowley JJ, Moënne-Loccoz Y, Dowling DN, Bruijn S, O'Gara F (1997). Biological control of *Pythium ultimum* by *Stenotrophomonas maltophilia* W81 is mediated by an extracellular proteolytic activity. Microbiology..

[55] Shin D-S, Park M-S, Jung S-R, Lee M-S, Lee K-H, Bae K-S, Kim S-B (2007). Plant growth-promoting potential of endophytic bacteria isolated from roots of coastal sand dune plants. J Microbiol Biotechnol..

[56] Mavrodi OV, Walter N, Elateek S, Taylor CG, Okubara PA (2012). Suppression of *Rhizoctonia* and *Pythium* root rot of wheat by new strains of *Pseudomonas*. Biol Control..

[57] Campos SB, Lisboa BB, Camargo FAO, Bayer C, Sczyrba A, Dirksen P, Albersmeier A, Kalinowski J, Beneduzi A, Costa PB, Passaglia LMP, Vargas LK, Wendisch VF (2016). Soil suppressiveness and its relations with the microbial community in a Brazilian subtropical agroecosystem under different management systems. Soil Biol Biochem..

[58] Xiong W, Li R, Ren Y, Liu C, Zhao Q, Wu H, Jousset A, Shen Q (2017). Distinct roles for soil fungal and bacterial communities associated with the suppression of vanilla *Fusarium* wilt disease. Soil Biol Biochem..

[59] De Corato U, Patruno L, Avella N, Salimbeni R, Lacolla G, Cucci G, Crecchio C (2020). Soil management under tomato-wheat rotation increases the suppressive response against *Fusarium* wilt and tomato shoot growth by changing the microbial composition and chemical parameters. Appl Soil Ecol..

[60] Aliyu H, Gorte O, de Maayer P, Neumann A, Ochsenreither K (2020). Genomic insights into the lifestyles, functional capacities and oleagenicity of members of the fungal family Trichosporonaceae. Sci Rep..

[61] Bonilla N, Gutiérrez-Barranquero JA, de Vicente A, Cazorla FM (2012). Enhancing soil quality and plant health through suppressive organic amendments. Diversity..

[62] Saison C, Degrange V, Oliver R, Millard P, Commeaux C, Montange D, Le Roux X (2006). Alteration and resilience of the soil microbial community following compost amendment: effects of compost level and compost-borne microbial community. Environ Microbiol..

