# Supplementary material for: Soil Suppressiveness Against Pythium ultimum and Rhizoctonia solani in Two Land Management Systems and Eleven Soil Health Treatments
Source: Microb Ecol. 2023 Mar 31;86(3):1709–24. doi: 10.1007/s00248-023-02215-9 (PMC10497426; doi:10.1007/s00248-023-02215-9)
Supplement: Supplementary file 1 — ESM 1 [file 248_2023_2215_MOESM1_ESM.docx]

**Supplementary information**

**Soil suppressiveness against *Pythium ultimum* and *Rhizoctonia solani* in two land management systems and eleven soil health treatments**

Viola Kurm^1*^, Johnny Visser^2^, Mirjam Schilder^1^, Els Nijhuis^1^, Joeke Postma^1^ & Gerard Korthals^1^

^1^Wageningen University and Research, Biointeractions and Plant Health, P.O. Box 16, 6700 AA Wageningen, The Netherlands

^2^Wageningen University and Research, Field Crops, Edelhertweg 1, 8219 PH Lelystad, The Netherlands

*e-mail: viola.kurm@wur.nl

**Table S1** Mean annual climate data in the years 2018 and 2019 at the site of the “Soil Health Experiment” in Vredepeel, The Netherlands.

| **Year** | **Annual precipitation (mm)** | **Average annual temperature (°C)** | **Average minimum temperature (°C)** | **Average maximum temperature (°C)** |
| --- | --- | --- | --- | --- |
| **2018** | **541** | **12.5** | **7.4** | **17.7** |
| **2019** | **663** | **12.3** | **7.3** | **17.2** |

**Table S2** Average of chemical soil properties for the two landmanagement systems and the different treatments measured in 2019.

| **System** | **Treatments** | **Total N (mg N/kg)** | **pH** | **Organic matter (%)** | **CEC (mmol+/kg)** | **C/N** | **Sand (%)** | **Silt (%)** | **Anorganic C (%)** | **Organic C (%)** |
| --- | --- | --- | --- | --- | --- | --- | --- | --- | --- | --- |
| **Organic** | **AHC** | **1687.50** | **5.53** | **4.20** | **65.50** | **14.25** | **88.25** | **7.50** | **0.03** | **2.43** |
| **Organic** | **ASD** | **1110.00** | **5.40** | **3.75** | **55.25** | **19.50** | **89.00** | **7.50** | **0.03** | **2.20** |
| **Organic** | **CHI** | **1080.00** | **5.50** | **3.70** | **63.50** | **19.00** | **88.75** | **7.50** | **0.03** | **2.05** |
| **Organic** | **CMP** | **1170.00** | **5.45** | **3.98** | **65.75** | **19.25** | **87.00** | **8.75** | **0.03** | **2.25** |
| **Organic** | **CTR** | **1112.50** | **5.45** | **3.53** | **56.50** | **17.75** | **87.50** | **9.25** | **0.03** | **2.00** |
| **Organic** | **GCC** | **1055.00** | **5.45** | **3.48** | **55.00** | **19.00** | **87.75** | **8.75** | **0.03** | **2.03** |
| **Organic** | **HRM** | **1230.00** | **4.98** | **3.85** | **47.25** | **17.50** | **89.00** | **6.75** | **0.04** | **2.18** |
| **Organic** | **MIX** | **1172.50** | **5.40** | **3.85** | **57.00** | **18.25** | **88.50** | **7.75** | **0.03** | **2.20** |
| **Organic** | **OSD** | **1175.00** | **5.33** | **4.05** | **62.50** | **19.25** | **88.25** | **7.25** | **0.03** | **2.30** |
| **Organic** | **TAG** | **1157.50** | **5.48** | **3.78** | **59.00** | **18.50** | **88.75** | **7.25** | **0.03** | **2.18** |
| **Conventional** | **AHC** | **1390.00** | **5.40** | **4.03** | **62.00** | **16.50** | **87.00** | **8.25** | **0.03** | **2.30** |
| **Conventional** | **ASD** | **1112.50** | **5.40** | **3.65** | **54.25** | **19.25** | **88.75** | **7.25** | **0.03** | **2.13** |
| **Conventional** | **CHI** | **1182.50** | **5.50** | **3.70** | **63.50** | **17.75** | **89.00** | **6.75** | **0.03** | **2.10** |
| **Conventional** | **CMP** | **1165.00** | **5.30** | **3.95** | **58.25** | **19.75** | **87.75** | **7.50** | **0.04** | **2.25** |
| **Conventional** | **CSD** | **992.50** | **5.28** | **3.55** | **52.25** | **20.75** | **88.75** | **7.50** | **0.04** | **2.05** |
| **Conventional** | **CTR** | **1060.00** | **5.55** | **3.60** | **56.00** | **19.50** | **88.00** | **8.50** | **0.04** | **2.05** |
| **Conventional** | **GCC** | **1075.00** | **5.40** | **3.68** | **54.75** | **20.00** | **89.00** | **6.75** | **0.03** | **2.18** |
| **Conventional** | **HRM** | **1215.00** | **5.05** | **3.88** | **49.00** | **19.25** | **88.25** | **8.00** | **0.03** | **2.33** |
| **Conventional** | **MIX** | **1115.00** | **5.25** | **3.78** | **55.50** | **20.25** | **87.00** | **8.75** | **0.03** | **2.28** |
| **Conventional** | **TAG** | **1070.00** | **5.25** | **3.68** | **55.25** | **20.00** | **88.50** | **7.25** | **0.04** | **2.18** |


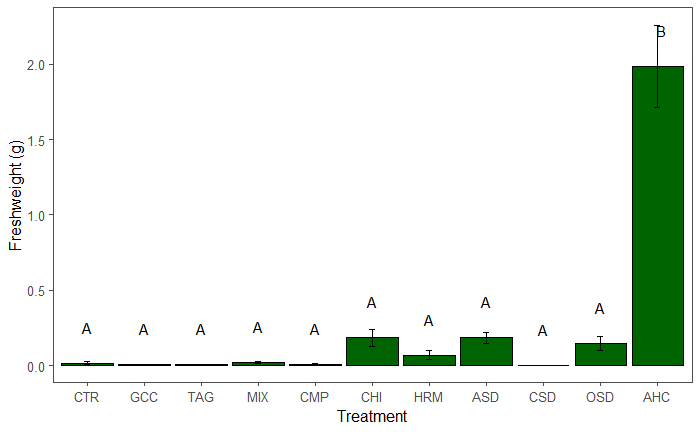


**Fig. S1** Average fresh weight of garden cress after inoculation with *P. ultimum* in 2018 in the treatments; error bars represent the standard error; different letters indicate significant differences between the treatments (p<0.05)


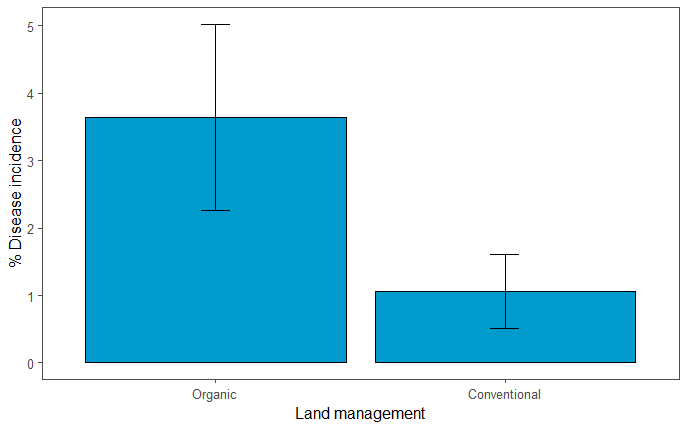


**Fig. S2** Average disease incidence for natural infection of *R. solani* in 2018 in the two land management systems; error bars represent the standard error.


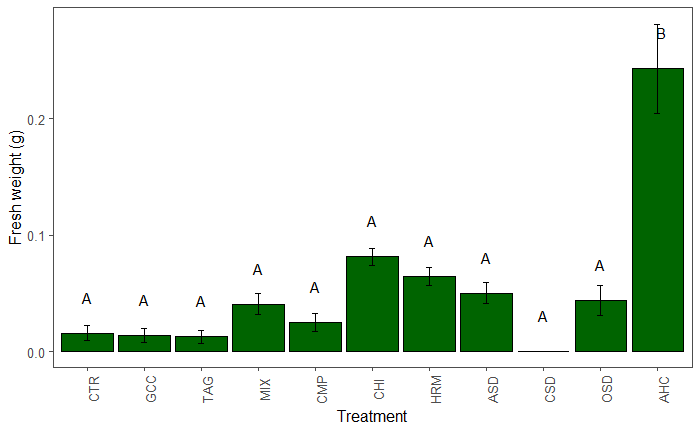


**Fig. S3** Average fresh weight of garden cress after inoculation with *P. ultimum* in 2019 in the treatments; error bars represent the standard error; different letters indicate significant differences between the treatments (p<0.05).


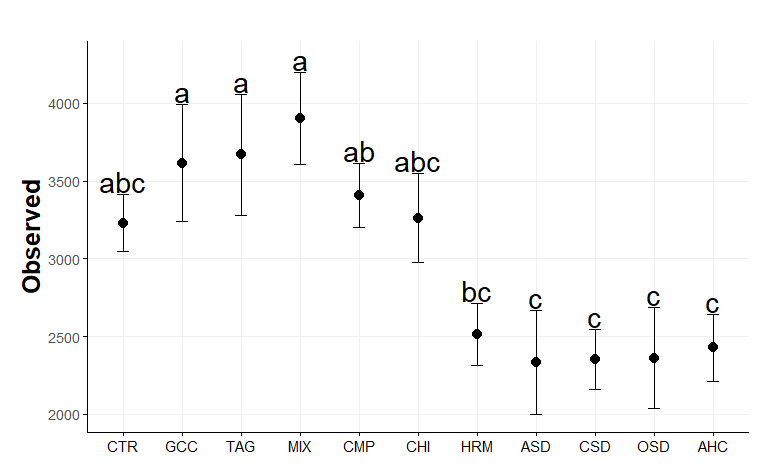


**Fig. S4** Observed diversity (i.e. OTU richness) of bacteria in the 11 treatments; different letters indicate significant differences between the treatments (p<0.05).

a


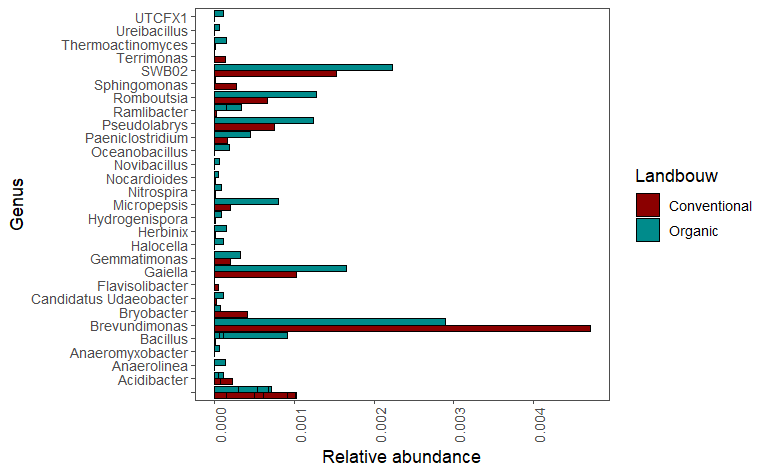


b


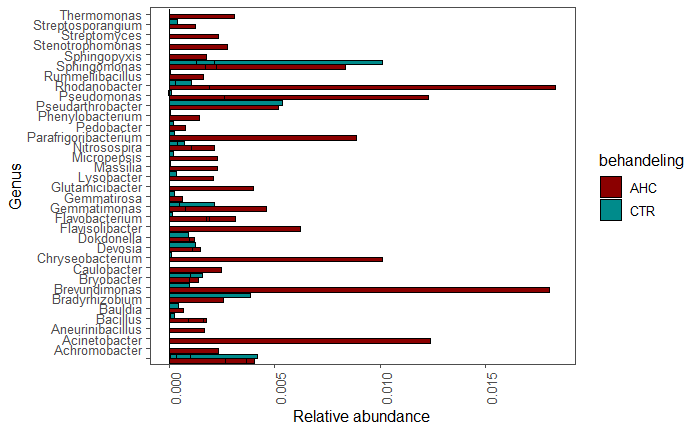


**Fig. S5** Relative abundance of bacterial genera significantly differing in abundance between a) conventional and organic agriculture and b) the CTR and AHC treatment.


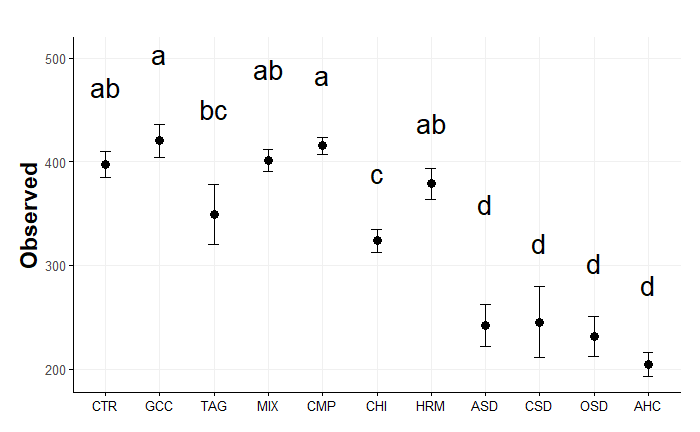


**Fig. S6** Observed diversity (i.e. OTU richness) of fungi in the 11 treatments; different letters indicate significant differences between the treatments (p<0.05).

a


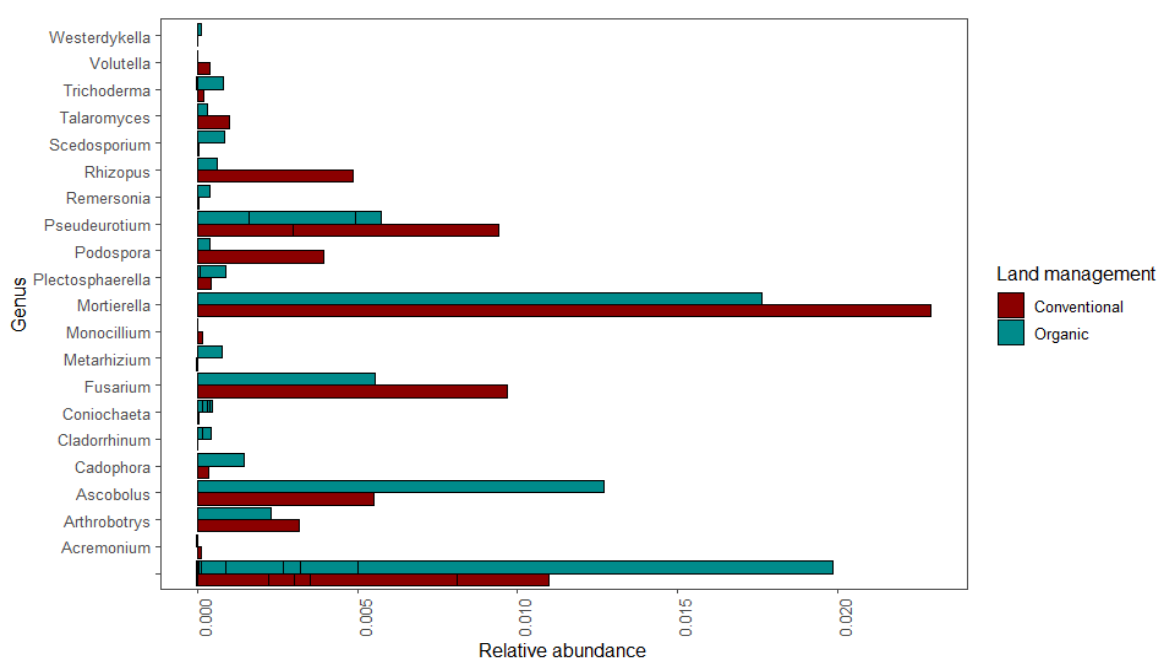


b


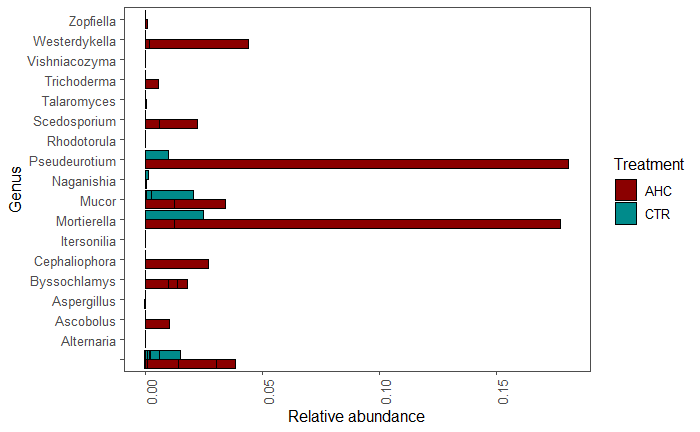


**Fig. S7** Relative abundance of fungal genera significantly differing in abundance between a) conventional and organic agriculture and b) the CTR and AHC treatment.


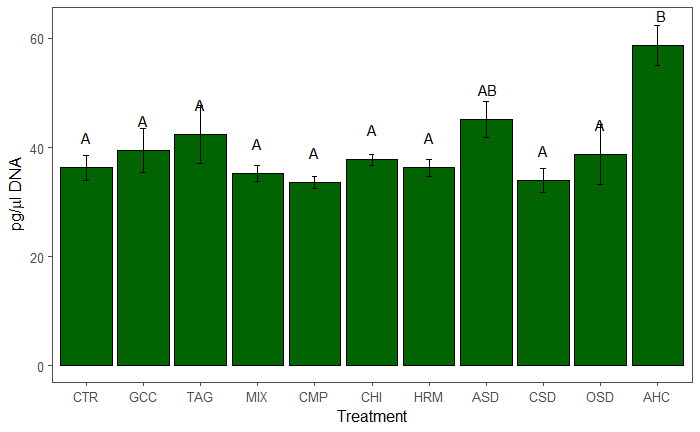


**Fig. S8** Average amount of bacterial DNA in the different treatments; error bars represent the standard error; different letters indicate significant differences between the treatments (p<0.05).


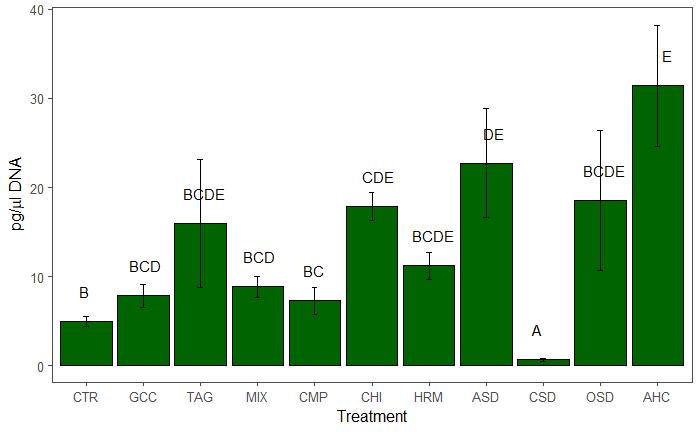


**Fig. S9** Average amount of fungal DNA in the different treatments; error bars represent the standard error; different letters indicate significant differences between the treatments (p<0.05).

**Table S3** Average crop yield of leek in 2019 in the two management systems and ten treatments.

| Management | Soil Health Treatment | Crop yield of leek |
| --- | --- | --- |
| Organic | CTR | 38.7 |
| Organic | GCC | 41.94 |
| Organic | TAG | 43.47 |
| Organic | MIX | 41.7 |
| Organic | ASD | 39.15 |
| Organic | CHI | 42.87 |
| Organic | OSD | 38.42 |
| Organic | CMP | 42.12 |
| Organic | HRM | 43.48 |
| Organic | AHC | 40.27 |
| Conventional | CTR | 43.45 |
| Conventional | GCC | 42.48 |
| Conventional | TAG | 42.41 |
| Conventional | MIX | 46.48 |
| Conventional | ASD | 46.08 |
| Conventional | CHI | 47.23 |
| Conventional | CSD | 44.06 |
| Conventional | CMP | 46.41 |
| Conventional | HRM | 47.27 |
| Conventional | AHC | 48.1 |
